# Changes in serum phosphate and potassium and their effects on mortality in malnourished African HIV-infected adults starting antiretroviral therapy and given vitamins and minerals in lipid-based nutritional supplements: secondary analysis from the Nutritional Support for African Adults Starting Antiretroviral Therapy (NUSTART) trial

**DOI:** 10.1017/S0007114517000721

**Published:** 2017-04-10

**Authors:** Andrea Mary Rehman, Susannah Louise Woodd, Douglas Corbett Heimburger, John Robert Koethe, Henrik Friis, George PrayGod, Lackson Kasonka, Paul Kelly, Suzanne Filteau

**Affiliations:** 1 London School of Hygiene & Tropical Medicine, London WC1E 7HT, UK; 2 Vanderbilt University Medical Center, Nashville, TN 37232, USA; 3 University of Copenhagen, 1958 Copenhagen, Denmark; 4 Mwanza Research Centre, National Institute for Medical Research, Mwanza, Tanzania; 5 University Teaching Hospital, Lusaka, Zambia; 6 Barts and the London School of Medicine, Queen Mary University of London, London E1 2AT, UK

**Keywords:** Potassium, Phosphate, HIV, Antiretroviral therapy, Malnutrition

## Abstract

Malnourished HIV-infected patients starting antiretroviral therapy (ART) are at high risk of early mortality, some of which may be attributed to altered electrolyte metabolism. We used data from a randomised controlled trial of electrolyte-enriched lipid-based nutritional supplements to assess the association of baseline and time-varying serum phosphate and K concentrations with mortality within the first 12 weeks after starting ART. Baseline phosphate results were available from 1764 patients and there were 9096 subsequent serum phosphate measurements, a median of 6 per patient. For serum K there were 1701 baseline and 8773 subsequent measures, a median of 6 per patient. Abnormally high or low serum phosphate was more common than high or low serum K. Controlling for other factors found to affect mortality in this cohort, low phosphate which had not changed from the previous time interval was associated with increased mortality; the same was not true for high phosphate or for high or low K. Both increases and decreases in serum electrolytes from the previous time interval were generally associated with increased mortality, particularly in the electrolyte-supplemented group. The results suggest that changes in serum electrolytes, largely irrespective of the starting point and the direction of change, were more strongly associated with mortality than were absolute electrolyte levels. Although K and phosphate are required for tissue deposition during recovery from malnutrition, further studies are needed to determine whether specific supplements exacerbate physiologically adverse shifts in electrolyte levels during nutritional rehabilitation of ill malnourished HIV patients.

The last decade has seen great advances in expanding access to antiretroviral therapy (ART) for HIV-infected Africans. However, poor survival in the first few months of ART remains a major concern and undernutrition is a consistent risk factor for this early mortality^(^ ^1^ ^–^ ^4^ ^)^. Malnutrition in these studies has usually been indicated by low BMI but mortality may result from metabolic alterations associated with low BMI, not simply from loss of lean or fat tissue^(^ ^5^ ^)^. Severe malnutrition is associated with disordered metabolism of Na, K, P and Mg^(^ ^6^ ^)^. Low serum phosphate is often present among malnourished Africans starting ART and is an independent risk factor for early mortality^(^ ^7^ ^)^.

We conducted the Nutritional Support for African Adults Starting Antiretroviral Therapy (NUSTART) trial to test if a high content of vitamins and minerals in a lipid-based nutritional supplement (LNS) would decrease mortality of malnourished adults referred for ART^(^ ^8^ ^)^. The intervention used a two-stage dietary protocol based on established methods for managing malnutrition among young children whereby there is a stabilisation phase providing micronutrients and electrolytes but only modest energy content followed by a rehabilitation phase with higher energy content in order to permit weight gain^(^ ^6^ ^)^. We focused on micronutrients because of their potential benefit for HIV patients,^(^ ^9^ ^)^ and on electrolytes, both because of their importance in treating malnutrition^(^ ^6^ ^)^ and because low phosphate in HIV patients starting ART may be similar to a potentially dangerous refeeding syndrome^(^ ^7^ ^)^.

In the NUSTART trial, the addition of a vitamin and mineral fortificant to the LNS (LNS-VM) did not decrease mortality within the first 12 weeks of ART compared with the unfortified LNS but did result in increases in some anthropometric measures and CD4 count (adjusted improvement of 25 cells/µl)^(^ ^8^ ^,^ ^10^ ^)^. The LNS-VM was also associated with increased incidence of both high phosphate (>1·45 mmol/l; rate ratio (RR) 1·23 compared with LNS) and high K (>5·5 mmol/l; RR 1·6), and decreased incidence of low phosphate (<0·65 mmol/l; RR 0·73)^(^ ^8^ ^)^. Previous work from our group has shown increased mortality associated with low serum phosphate (17 % increase/0·1 mmol/l increase)^(^ ^7^ ^)^; however, analysis of NUSTART patients found significant nonlinear associations between baseline electrolytes and survival and before starting ART, high, rather than low, phosphate appeared to carry a greater risk of death^(^ ^11^ ^)^. In addition, we observed that the LNS-VM decreased renal wasting of K and phosphate but not Mg^(^ ^12^ ^)^. In view of the effects of the LNS-VM intervention on electrolyte metabolism and on the association of electrolyte metabolism with mortality, we conducted a secondary analysis of NUSTART data. We performed detailed time course analysis of the effects of the intervention and its associated time-varying consequences on electrolyte levels and on the changes in electrolyte levels in relation to mortality in the trial.

## Methods

### Study design and intervention

NUSTART was an individually randomised, controlled, two-site, two-arm, phase III trial comparing LNS (Nutriset) either with (LNS-VM) or without (LNS) high levels of vitamins and minerals among participants who were HIV-positive and malnourished. The trial design, detailed intervention and primary findings have been reported previously^(^ ^8^ ^)^. The intervention was given at two graduated energetic doses mimicking nutritional management of malnourished children^(^ ^6^ ^)^; daily vitamin and mineral doses were the same in both the low-energy period given from referral to ART until 2 weeks after starting ART and the higher energy period given from weeks 2 to 6 of ART. Follow-up without supplementation continued until week 12 of ART. Varying actual ART start dates resulted in varied pre-ART durations of LNS or LNS-VM consumption with the median and mode being 3 weeks. The daily phosphate intake from the fortified LNS-VM was 38–47 mmol (according to the manufacturer’s analyses; the range is due to variability in product batches); for comparison, the UK upper reference nutrient intake (RNI) is 17·5 mmol/d for adults^(^ ^13^ ^)^. The daily K intake from LNS-VM was 30–32 mmol; the UK RNI for adults is 90 mmol/d. The trial was registered at PACTR201106000300631.

### Setting and participants

The trial was conducted between August 2011 and December 2013 at the National Institute for Medical Research (NIMR), Mwanza, Tanzania and the University Teaching Hospital, Lusaka, Zambia. Participants were recruited from HIV clinics near the two trial sites. Eligible participants had BMI <18·5 kg/m^2^, were ART naive (except for previous short course regimens used to prevent mother to child HIV transmission), needed to start ART as determined by CD4 count <350 cells/μl or WHO stage 3 or 4^(^ ^14^ ^)^, were at least 18 years old, willing to undertake intensive follow-up in the study clinics, and providing informed consent. Participants were ineligible if participating in a potentially conflicting research study or self-reported a pregnancy.

### Randomisation and masking

The statistician member of the independent data safety and monitoring committee generated the randomisation list. The list was computer generated in blocks of sixteen and stratified by country. Clinic pharmacists labelled the packets of LNS-VM and LNS with study ID numbers as packages were dispensed. Clinic nurses who were blinded to the randomisation list assigned sequential (within site) ID numbers.

### Follow-up and data management

Patients were seen weekly from recruitment until the ART initiation visit, then at 2, 4, 6, 8, and 12 weeks after starting ART. Participants could also come for unscheduled visits at any time.

At each visit, including unscheduled ones, medical and other examinations were conducted^(^ ^8^ ^)^ and blood samples were collected for electrolyte assays. For ethical reasons, we provided specific electrolyte supplements to participants with low electrolyte values. Phosphate tablets (97 mmol/d for 7 d) were supplied to thirty-seven participants and K tablets (48 mmol/d for 7 d) to nine participants. One participant in Lusaka received intravenous K (40 mmol over 8 h).

### Laboratory analysis

In Lusaka serum phosphate was measured spectrochemically on a Pointe 180 analyser (Pointe Scientific). Sample results were accepted only from runs for which the external quality control (QC) sample from the same supplier was within expected range; for unacceptable runs, samples were reanalysed where possible or omitted if not. Inter-assay CV for this external QC was 7 %. In Mwanza serum phosphate was measured in an external laboratory (Bugando Medical Centre) using a Roche COBAS Integra 400 analyser (Roche Molecular Diagnostics). Serum K was measured by optical emission using Perkin Elmer Optima 7000 ICP (Perkin Elmer). An external QC (Seronorm; Alere) was run each day and values were within expected limits at both sites. Inter-assay CV for K were 5 % in Mwanza and 6 % in Lusaka.

Cut-offs for low (<0·65 mmol/l) serum phosphate and low (<2·5 mmol/l) or high (>6·5 mmol/l) serum K levels were set according to the US National Institutes of Health, Division of AIDS (DAIDS^(^ ^15^ ^)^) grades 3 and 4. DAIDS does not set high ranges for phosphate so we considered as above normal limits if >1·45 mmol/l and below normal limits if <0·87 mmol/l^(^ ^16^ ^)^; for comparison we also looked at any K above, >5·5 mmol/l, or below, <3·5 mmol/l, normal limits^(^ ^16^ ^)^ which were equivalent to grades 1 or 2 DAIDS.

### Statistical analysis and outcomes

Data were double entered into OpenClinica data management system in Lusaka and into CSPro 4.1 and stored in MySQL databases in Mwanza. Analyses were conducted in STATA version 14.1.

Electrolyte levels at the end of follow-up (12 weeks post-ART) were analysed as continuous variables. Means were compared between treatment arms using *t* tests and linear regression adjusting for baseline values. Comparisons at the end of follow-up, by definition, used the subset of patients who survived and attended the 12-week visit within 14 d of the scheduled date (i.e. up to 14 weeks post-ART) as decided *a priori*. However, we also wanted to investigate treatment effects using all measured data, including data from patients who died or were lost to follow-up until the point they were lost from the study. We used piecewise mixed-effects cubic regression models with the time axis split at the date of starting ART. Random intercepts and random linear slopes were incorporated into the model. This model fitted two lines per person with differing slopes, restricting the lines to join at the date of starting ART. *P* values provide evidence of whether the shape of curves differed by treatment arm. For presentation, marginal predictions are graphed and are based on the median time, 21 d, spent before starting ART.

Knowing that LNS-VM was associated with increased incidence of high electrolyte values, and that risk of death before starting ART appeared higher with increasing levels of baseline serum phosphate^(^ ^11^ ^)^, we sought to examine the effect of the intervention on mortality allowing for the time-varying measurements of electrolyte values. Time was split into 4-week bands and also split pre- and post-starting ART, and controlled for in the analysis. Participants with electrolytes measured only once (*n* 156 for phosphate; *n* 104 for K) were excluded because time changes could not be determined, and those missing ART start date (*n* 31) were excluded from analysis because the split could not be performed. Participants were censored at 14 weeks after starting ART, at their last visit if they were lost to follow-up or withdrew, or at 10 weeks after recruitment for the 114 participants who had not yet started ART by this point. We used time-to-event shared frailty Poisson regression models to estimate the effect of treatment arm on mortality using hazard ratios, both unadjusted and adjusted for factors associated with mortality. The current (and time changing) value of the electrolyte was categorised into tertiles of high, middle or low. Standardised changes from the previous value, to control for varying intervals between visits, were categorised into increase, no change or decrease; for K increase or decrease was defined as >0·03 mmol/d and for phosphate it was defined as >0·02 mmol/d. These two variables were incorporated into models as main effects and additionally tested for interaction. When interaction was present, current level and change from previous level were combined to form a categorical variable with nine levels. In either case, these variables were allowed to vary over time in the model.

### Sample size

Recruitment to the trial was stopped early, following advice from the trial Data Safety and Monitoring Board, because a higher than expected mortality rate (not related to the intervention) meant we already had sufficient power for the primary outcome of mortality^(^ ^8^ ^)^. The 1815 participants who were enrolled in the trial gave 90 % power to detect a difference of 30 % between trial arms in the primary outcome of mortality. The 800 participants with electrolyte values measured at 12 weeks after initiating ART gave 80 % power to detect a difference of 0·20 mmol/l between trial arms.

### Ethics

The study was conducted according to the guidelines laid down in the Declaration of Helsinki and all procedures involving human participants were approved by the ethics committee of the London School of Hygiene and Tropical Medicine, the Medical Research coordinating committee of NIMR, Tanzania and the University of Zambia Biomedical Research Ethics Committee. Written or thumbprint consent was obtained from all participants. Patients received medical care from local health services according to national guidelines.

## Results

Participants were comparable at baseline between trial arms (Table 1 for participants with phosphate data and the online Supplementary Table S1 for the slightly different number of participants with K data). Of 1815 participants, 1764 (97 %) had baseline serum phosphate data and 1710 (94 %) had baseline K data (Table 2). Phosphate at baseline was above normal levels (>1·45 mmol/l) in one-fifth of participants and below normal (<0·87 mmol/l) in 14 %; K was above normal levels (>5·5 mmol/l) in 3 % and below normal (<3·5 mmol/l) in 15 % of participants (Table 2). Only 3·4 % had low levels of both K (<3·5 mmol/l) and phosphate (<0·87 mmol/l) at baseline.

**Table 1:** Baseline characteristics of patients included in the evaluation of the effect of Nutritional Support for African Adults Starting Antiretroviral Therapy intervention on serum phosphate (Numbers and percentages; mean values and standard deviations)

	LNS (*n* 883)		LNS-VM (*n* 898)	
	*n*	%	*n*	%
Age (years)				
Mean	35·8		35·9	
sd	9·44		9·33	
Female	445	50·4	437	48·7
On tuberculosis treatment*	129	14·6	173	19·3
Baseline CD4 count (cells/µl)				
Mean	140·0		135·6	
sd	103·3		96·7	
CD4 count <100 (cells/µl)	383	43·4	385	42·9
BMI <17 (kg/m^2^)	523	59·2	528	58·8
Social economic status				
Lowest	193	21·9	166	18·5
Low	184	20·8	181	20·2
Middle	160	18·1	194	21·6
High	180	20·4	170	18·9
Highest	166	18·8	187	20·8
Marital status*				
Married	430	48·7	411	45·8
Widow/widower	104	11·8	95	10·6
Divorced/separated	237	26·8	261	29·1
Single	108	12·2	129	14·4
Lives with partner	3	0·3	2	0·2
Occupation*				
Salaried	135	15·3	130	14·5
Self-employed	454	51·4	475	52·9
Housewife	93	10·5	84	9·4
Student	9	1·0	9	1·0
Unemployed	191	21·6	200	22·3
Education level*				
None	167	18·9	168	18·7
Primary	508	57·5	518	57·7
Secondary	184	20·8	188	20·9
University/tertiary	23	2·6	24	2·7
Study site				
Lusaka	540	61·2	552	61·5
Mwanza	343	38·8	346	38·5

LNS, lipid-based nutritional supplement; LNS-VM, lipid-based nutritional supplement with added vitamins and minerals.

* Missing values for: on tuberculosis treatment, 7 (0·8 %) LNS arm, 6 (0·7 %) LNS-VM arm; marital status: 1 (0·1 %) LNS arm; occupation: 1 (0·1 %) LNS arm; education level: 1 (0·1 %) LNS arm.

**Table 2:** Serum phosphate and potassium values at baseline among patients randomised to lipid-based nutritional supplement (LNS) or lipid-based nutritional supplement with added vitamins and minerals (LNS-VM) (Mean values and standard deviations; numbers and percentages)

	LNS-VM		LNS	
	*n* *	%	*n* *	%
Serum phosphate (mmol/l)	888		876	
Mean	1·24		1·24	
sd	0·41		0·37	
<0·65 mmol/l†	27	3	24	3
<0·87 mmol/l‡	113	12	83	9
>1·45 mmol/l‡	177	20	169	19
Missing	26	3	25	3
Serum K (mmol/l)	861		849	
Mean	4·14		4·09	
sd	0·73		0·72	
<2·5 mmol/l†	10	1	14	2
<3·5 mmol/l‡	133	15	141	16
>5·5 mmol/l‡	27	3	16	2
>6·5 mmol/l†	7	0·8	2	0·2
Missing	53	6	52	6

* Total enrolled in the trial were: LNS-VM arm, *n* 914; LNS arm, *n* 901.

† Values from Division of AIDS grades 3 or 4 adverse events^(^ ^15^ ^)^.

‡ Values below or above normal range^(^ ^16^ ^)^.

Up to 14 weeks after starting ART, there were 9096 subsequent phosphate results in 1608 participants, median 6 (interquartile range (IQR) 3–8) per patient, and 8773 subsequent K results in 1606 participants, median 6 (IQR 3–7) per patient, with comparable numbers of observations in each trial arm. Above normal phosphate (>1·45 mmol/l) was the most commonly occurring abnormal electrolyte result (Table 3). Episodes of above normal phosphate measured on consecutive occasions, that is weekly, every 2 or every 4 weeks, depending on the time before or after starting ART, occurred in 368 (204 from LNS-VM arm) participants for a median episode length of 14 (IQR 7–28) d. Episodes of low phosphate (<0·65 mmol/l; DAIDS grades 3 and 4) measured on consecutive occasions occurred in twenty-eight participants for a median episode length of 14 (IQR 7–17·5) d. There were no occasions where high K (>6·5 mmol/l; DAIDS grades 3 and 4) was measured on consecutive occasions. Seven participants experienced episodes of K remaining above normal, 5·5 mmol/l (equivalent to DAIDS grades 1 and 2), for a median of 7 (IQR 7–14) d and eleven participants had low K (<2·5 mmol/l; DAIDS grades 3 and 4) for a median of 14 (IQR 7–38) d.

**Table 3:** Abnormal serum electrolyte values over the study period by trial arm and total in study (Numbers of measurements (*N*_m_) and number of participants (*N*_p_))

			Pre-ART*				Post-ART					
	Total		Post baseline to 21 d		>21 d		ART to 14 d after ART		15–42 d after ART		43–98 d after ART†	
Adverse event or abnormality	*N*_m_	*N*_p_	*N*_m_	*N*_p_	*N*_m_	*N*_p_	*N*_m_	*N*_p_	*N*_m_	*N*_p_	*N*_m_	*N*_p_
Phosphate <0·65 mmol/l‡												
LNS-VM	106	87	17	14	7	6	22	17	30	25	30	24
LNS	137	113	42	37	3	3	42	31	28	24	23	18
Phosphate >1·45 mmol/l§												
LNS-VM	1081	514	353	194	89	28	207	111	204	85	228	96
LNS	834	432	227	130	65	25	142	69	177	86	223	122
*n* in study												
LNS-VM	4717	816	1285	716	389	218	980	623	1008	569	1055	525
LNS	4379	792	1232	701	284	170	971	598	935	538	957	491
K <2·5 mmol/l‡												
LNS-VM	38	28	9	7	8	4	7	5	7	5	7	7
LNS	43	36	17	15	3	2	7	5	8	8	8	6
K >6·5 mmol/l‡												
LNS-VM	33	32	8	8	4	3	9	9	5	5	7	7
LNS	19	19	5	5	2	2	5	5	1	1	6	6
K >5·5 mmol/l‡												
LNS-VM	132	114	50	44	15	11	29	26	19	17	19	16
LNS	77	71	30	30	11	8	13	12	10	10	13	11
*n* in study												
LNS-VM	4542	811	1249	704	368	213	949	597	979	557	997	523
LNS	4231	795	1203	693	271	166	939	578	908	526	910	487

ART, antiretroviral therapy; LNS, lipid-based nutritional supplement; LNS-VM, lipid-based nutritional supplements fortified with vitamins and minerals.

* Individuals varied in the time spent in the pre-ART period, median 3 (interquartile range 2·1–4·7) weeks.

† This period was after supplementation ended.

‡ Division of AIDS grades 3 or 4^(^ ^15^ ^)^.

§ Above normal limits^(^ ^16^ ^)^.

At the end of follow-up, 6 weeks after discontinuation of the study supplement, there was no evidence of a difference between trial arms in mean serum phosphate (overall mean 1·25 (sd 0·35) mmol/l), adjusted difference 0·03 (95 % CI −0·02, 0·08, *P*=0·24, *n* 798) or mean serum K (overall mean 4·13 (sd 0·64) mmol/l), adjusted difference 0·002 (95 % CI −0·10, 0·10, *P*=0·97, *n* 754). Participants who had remained in the study and had electrolytes measured at the end of follow-up were older, more likely to be female, be on tuberculosis treatment at baseline, had higher socio-economic status, higher baseline BMI, higher baseline CD4 count and were less likely to be divorced; for having a phosphate measurement they were also more likely to be Zambian (online Supplementary Tables S2 and S3).

**Fig. 1:**
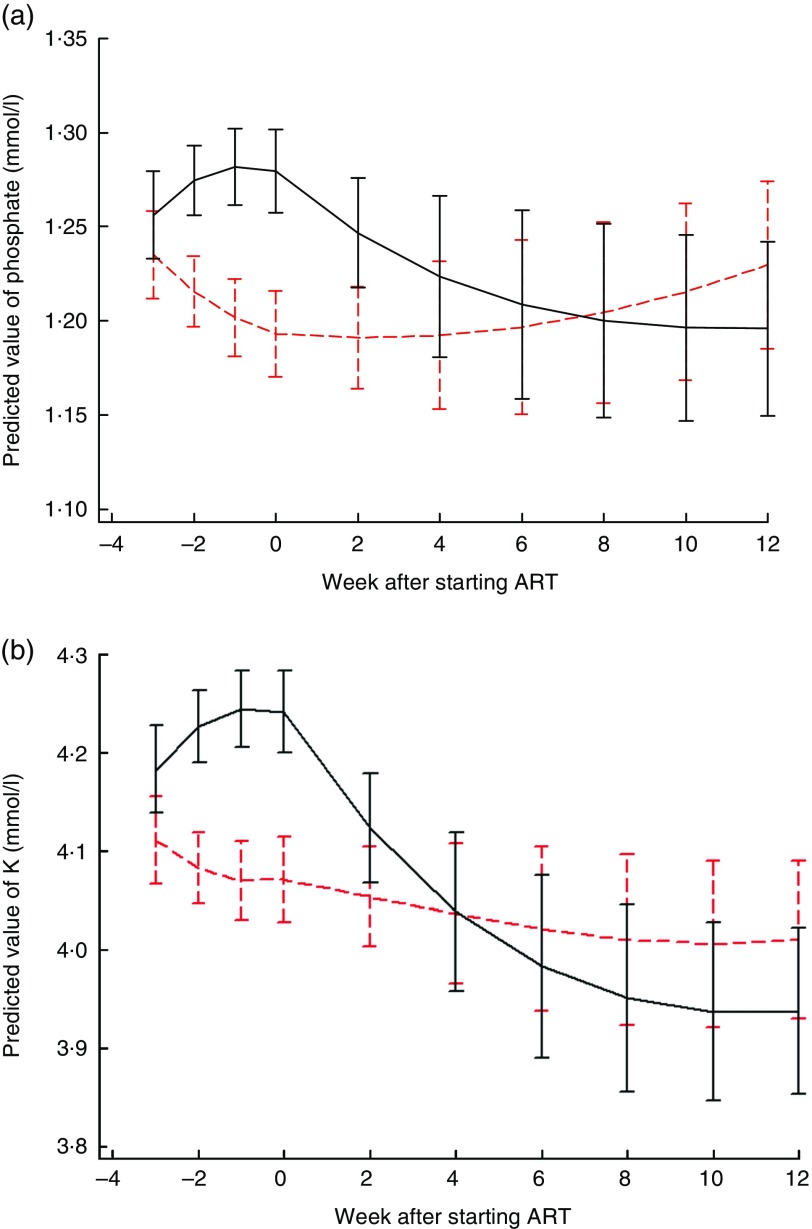
Changes in predicted mean serum electrolytes over time by trial arm, for an individual who started antiretroviral therapy (ART) 3 weeks after recruitment. (a) Phosphate: *P* values for differences between lipid-based nutritional supplement (LNS) and lipid-based nutritional supplements fortified with vitamins and minerals (LNS-VM) groups were 0·0002 overall, 0·008 pre-ART and 0·0003 post-ART. Curves represent marginal predictions based on all available data for all patients and are derived from piecewise cubic equations with random slopes and intercepts. Prediction equations have different slopes pre- and post-starting ART. (b) K: *P* values for differences between LNS and LNS-VM groups were 0·0002 overall, 0·0002 pre-ART and 0·004 post-ART. Curves represent marginal predictions based on all available data for all patients and are derived from piecewise cubic equations with random slopes and intercepts. Prediction equations have different slopes pre- and post-starting ART. 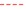, LNS; 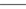, LNS-VM.

Fig. 1 shows electrolyte changes over time for patients who started ART at the median of 3 weeks after referral for treatment. Similar figures for participants who survived until the end of the study are shown in the online Supplementary Fig. S1 and S2, and for those starting ART 1, 2 or 4 weeks after referral are shown in the online Supplementary Fig. S3 and S4. There was strong evidence that changes in phosphate and K over time differed by trial arm; *P* values for differences between LNS and LNS-VM groups for both K and phosphate were 0·0002 overall and differences in the pre- and post-ART time periods were also highly significant (Fig. 1). In the LNS-VM arm values of electrolytes were found on average to increase up to the time ART started and then reduce to baseline levels by the end of follow-up whereas in the LNS arm average values changed little over follow-up time.

Mortality was higher among males, Tanzanians, those with oedema at baseline, those not on tuberculosis medication at baseline, and increased with age, with higher C-reactive protein (CRP), with lower CD4 cell count and with lower BMI at baseline (data presented by Woodd *et al*.^(^ ^11^ ^)^). The current value of plasma electrolyte and the change from the previous value showed significant interaction for phosphate analyses so the nine level categorical analysis is presented; these interactions were not significant for K, so main effects are presented (Table 4). Once adjusted for factors associated with mortality, there was some evidence overall (*P*=0·03, phosphate, *P*=0·06 K) that time-varying electrolytes affected mortality (Table 4, *n* 9027 phosphate measurements on 1588 participants, *n* 8709 K measurements on 1588 participants; median number of measurements in those dying was 3 (IQR 2, 5) and in those completing follow-up was 8 (IQR 6, 9)). Compared with participants with stable measurements around the mean, participants with electrolyte values which were not stable, had increased hazard of mortality (Table 4). Although there was no evidence that the effect of serum electrolytes was modified by trial arm (*P*_for interaction_>0·34), due to the LNS-VM arm containing electrolytes by design, we *a priori* also examined the effect of changes in electrolytes stratified by trial arm (online Supplementary Tables S4(a) and (b)); the estimates were comparable to those in Table 4. The effect of changes in phosphate was statistically significant in both arms, but the magnitudes of the adjusted hazard ratios tended to be greater in the LNS-VM than in the LNS arm. It should be noted that the intervention had no overall effect on mortality^(^ ^8^ ^)^ and that the number of deaths in some electrolyte groups was small in these analyses of separate trial arms.

**Table 4:** Poisson regression models for time-varying serum electrolyte values associated with mortality between referral for antiretroviral therapy (ART) and ART initiation (Unadjusted and adjusted hazard ratios (HR) and 95 % confidence intervals)

	Change from			Mortality rate/100 person-years							
Current values*	previous value†	Deaths (*n*)	100 person-years	HR‡	95 % CI	Unadjusted HR‡	95 % CI	*P*	Adjusted HR§	95 % CI	*P*
Phosphate||											
Low	Decrease	24	0·34	70	47, 105	3·59	2·15, 5·98		1·93	1·13, 3·32	
Low	No change	20	0·48	42	27, 65	1·82	1·06, 3·14		1·79	1·01, 3·17	
Low	Increase	4	0·02	180	68, 481	13·01	4·60, 36·75		7·09	2·34, 21·54	
Middle	Decrease	31	0·35	89	63, 127	4·89	3·04, 7·88		2·80	1·69, 4·67	
Middle	No change	38	1·67	23	17, 31	Ref.		<0·001	Ref.		<0·001
Middle	Increase	27	0·28	98	67, 142	4·65	2·82, 7·69		2·62	1·53, 4·49	
High	Decrease	5	0·05	107	45, 258	4·55	1·77, 11·65		3·75	1·39, 10·12	
High	No change	14	0·42	33	20, 56	1·43	0·78, 2·65		1·34	0·69, 2·61	
High	Increase	28	0·39	72	50, 104	3·27	2·00, 5·35		1·99	1·18, 3·35	
K¶											
Low	–	93	0·91	102	83, 125	2·09	1·55, 2·83		1·26	0·92, 1·73	
Middle	–	104	2·80	37	31, 45	Ref.		<0·001	Ref.		0·20
High	–	53	0·79	67	52, 88	1·52	1·06, 2·17		1·32	0·91, 1·92	
–	Decrease	97	0·94	103	84, 126	2·72	1·98, 3·74		1·48	1·06, 2·07	
–	No change	78	2·60	30	24, 37	Ref.		<0·001	Ref.		0·06
–	Increase	73	0·91	81	64, 101	2·36	1·67, 3·34		1·30	0·92, 1·85	

Ref., referent values.

* For phosphate low is 0·04–0·96, middle is 0·97–1·44, high is 1·45–5·27. For K low is 0·1–3·63, middle is 3·63–4·5 and high is 4·6–10 mmol/l.

† For serum phosphate daily changes were defined as: <−0·02 a decrease, (−0·02, 0·02) no change and >0·02 increase· For K daily changes were defined as <−0·03 a decrease, (−0·03, 0·03) no change and >0·03 an increase.

‡ A Lexis expansion for time was adjusted for in 4-week time bands, both pre-art and post-art. Being on ART was also adjusted for.

§ Adjusted for trial arm (lipid-based nutritional supplement or lipid-based nutritional supplements fortified with vitamins and minerals), time band (as for ‡), country, sex, age group (18–29, 30–39, 40–49, ≥50 years), baseline CD4 count (<50, 50–99, 100–199, ≥200 cells/μl), BMI (continuous), baseline tuberculosis treatment (yes/no), baseline oedema (yes/no), baseline C-reactive protein (<10, 10–49, 50–159, ≥160 mg/l).

|| *P*_for interaction_ value between current level and change from previous level unadjusted model=0·03, adjusted model=0·03.

¶ *P*_for interaction_ value between current level and change from previous level unadjusted model=0·85, adjusted model=0·47.

## Discussion

Our previous analysis of baseline serum electrolyte data and subsequent mortality in the malnourished NUSTART cohort^(^ ^11^ ^)^ showed no significant effects of baseline serum phosphate or K on mortality to 12 weeks after starting ART but this finding differed from the significant mortality risk of low phosphate at start of ART reported in a similar cohort of malnourished Zambians starting ART^(^ ^7^ ^)^. Given these conflicting findings regarding electrolyte levels at the time of treatment initiation, the present analysis extends these findings to time intervals both before and after starting ART in order to give a fuller picture of the dynamic situation in which serum electrolytes change over time under the influence of several factors: diet, tissue catabolism or anabolism, ART and renal function. The values in the LNS group may reflect the usual situation without provision of high levels of vitamins and minerals since, even though LNS is not usually given, food intake increases as health and appetite improve with ART^(^ ^17^ ^)^. The LNS-VM group shows the added effect of providing vitamins and minerals. High levels of serum K were uncommon at both baseline and during the intervention whereas high phosphate was common throughout, especially in the LNS-VM group. The level of phosphate in the LNS-VM preparation was about twice the RNI and much lower than the tolerable upper level^(^ ^13^ ^)^. Therefore the high incidence of above-normal serum phosphate suggests that malnourished, HIV-infected adults have much lower metabolic capacity to handle this electrolyte than do healthy individuals. In a small subset of the NUSTART Lusaka patients we did find evidence of abnormal renal wasting of electrolytes which was improved in the LNS-VM group by week 12^(^ ^12^ ^)^. Renal wasting of electrolytes would be expected to result in abnormally low, not high electrolytes. Unfortunately, we do not have data on renal electrolyte handling at any time points between baseline and 12 weeks ART.

Important questions are whether transient changes in serum electrolytes resulted in any adverse effects to the patients and whether relatively high intakes of electrolytes affected the number of adverse events. The results suggest that changes in serum electrolytes between visits, largely irrespective of the starting point and the direction of change, were more strongly associated with mortality than were absolute electrolyte levels. The changes appeared to have their greatest effects for phosphate, and high supplementary amounts of electrolytes through LNS-VM treatment may have accentuated the effects. Labile serum electrolyte levels support the idea of poor metabolic control among this malnourished population with advanced HIV, and high supplementary electrolytes may further impair this control. The lack of association between serum K and mortality may appear surprising. We were able to examine only the effect (and showed no association) of above average values, as the number of patients with abnormal, and in particular extremely abnormal values was low.

The study has several limitations. We are unable to comment on additional dietary sources of electrolytes as detailed dietary intakes were not measured in the trial. In addition, levels of phosphate in the supplements were analysed by the manufacturer which is a potential limitation, although likely not a large one as the two supplements were clearly different and the focus of the study was on patient metabolism, not intake. Although randomisation and a large sample size should have ensured balance between trial arms and reduced potential effects of residual confounding, in this observational investigation we cannot estimate causal associations between serum electrolyte levels and mortality. It is possible that electrolyte changes could also be a consequence of illness which leads to increased mortality through another causal pathway. We adjusted for illness, as measured by serum CRP and tuberculosis infection (treatment), although we know that some mortality could be partially explained by undiagnosed tuberculosis infection among other causes. In addition, by including electrolyte levels as a time-varying confounder we are making the assumption that the effect of electrolytes on mortality is not modified by follow-up time. An alternative formulation to investigate for time-modified confounding could be structural equation models. As our follow-up time was relatively short, our assumption, and simpler model, should be adequate. Finally, we were unable to assess for any time-varying influence of CD4 cell count as this was only measured at baseline and end of study which, if it influenced the relationship between electrolyte levels and mortality, could increase the potential for residual confounding.

The main strength of our study was the large longitudinal sample size of serum electrolyte values which were repeated at fairly regular intervals, weekly or fortnightly for most participants. In addition, being able to compare within participants from referral until after ART commenced provided valuable and clinically relevant information on changes over time. We, however, cannot rule out that visit frequency and timing could have been related in part to participant health, with both increases in frequency (unscheduled visits and extra serum testing due to illness) and decreases in frequency (very ill leading to death) occurring because of ill health. Given the large sample size, we would envisage that the results are robust enough to any form of systematic bias. We have chosen to present results combined over intervention groups partly because the intervention did not significantly affect mortality, but also because we know that participant adherence to supplements was on the low side, with only 39 % of participants consuming at least 75 % of their LNS sachets, thus reducing differences in electrolyte intake^(^ ^8^ ^)^. As we were not able to tell if adherence changed when the energetic dose increased at 2 weeks from ART initiation, we are not able to infer if the decrease in serum electrolyte levels post-ART could be partially explained by decreased dosing if participants were unable to consume all of their LNS at the higher energy level or if it was truly due to metabolic stabilisation or commencing ART.

Low serum phosphate is commonly seen in malnourished patients, both with and without HIV infection, and is associated with poor outcomes^(^ ^7^ ^,^ ^18^ ^,^ ^19^ ^)^. Our analyses focused on electrolyte metabolism, not dietary intake other than the differences between the LNS and LNS-VM preparations. However, dietary management of these patients remains critical and the amounts of phosphate and other electrolytes which should be provided in therapeutic diets for malnourished people in order to restore metabolic stability, increase tissue deposition and avoid the refeeding syndrome or other adverse effects is still under research^(^ ^19^ ^)^. Based on our results, we recommend that future interventions to improve nutritional status of malnourished HIV-infected patients should not include high doses of electrolytes early after starting ART as malnourished HIV patients appear unable to handle electrolytes, particularly phosphate, adequately. Once metabolism is stabilised and health improves, both K and phosphate are needed for tissue deposition so should be provided in adequate amounts.

## Supplementary material

For supplementary material accompanying this paper visit http://dx.doi.org/10.1017/S0007114517000721. click here to view supplementary material
