# Supplementary material for: Changes in serum phosphate and potassium and their effects on mortality in malnourished African HIV-infected adults starting antiretroviral therapy and given vitamins and minerals in lipid-based nutritional supplements: secondary analysis from the Nutritional Support for African Adults Starting Antiretroviral Therapy (NUSTART) trial
Source: Br J Nutr. 2017 Apr 10;117(6):814–21. doi: 10.1017/S0007114517000721 (PMC5426318; doi:10.1017/S0007114517000721)
Supplement: Supplementary file 1 [file S0007114517000721sup001.docx]

| Table S1. Baseline characteristics of patients included in the evaluation of the effect of NUSTART intervention on serum potassium | | |
| --- | --- | --- |
|  | LNS (n=886)^1^ | LNS-VM (n=900)^2^ |
| Age years, mean (SD^3^) | 35.8 (9.43) | 36.0 (9.39) |
| Female, n (%) | 449 (50.7%) | 437 (48.6%) |
| On tuberculosis treatment, n (%)^4^ | 129 (14.6%) | 172 (19.1%) |
| Baseline CD4 count cells/ µL, mean (SD^3^) | 140.0 (103.4) | 135.3 (96.8) |
| CD4 count <100 (cells/µL), n (%) | 385 (43.5%) | 388 (43.1%) |
| BMI <17 (kg/m^2^), n (%) | 525 (59.3%) | 531 (59.0%) |
| Social economic status, n (%) |  |  |
| Lowest | 194 (21.9%) | 166 (18.4%) |
| Low | 187 (21.1%) | 182 (20.2%) |
| Middle | 160 (18.1%) | 195 (21.7%) |
| High | 181 (20.4%) | 170 (18.9%) |
| Highest | 164 (18.5%) | 187 (20.8%) |
| Marital status, n (%)^4^ |  |  |
| Married | 431 (48.7%) | 412 (45.8%) |
| Widow/widower | 104 (11.7%) | 95 (10.6%) |
| Divorced/separated | 240 (27.1%) | 263 (29.2%) |
| Single | 107 (12.1%) | 128 (14.2%) |
| Lives with partner | 3 (0.3%) | 2 (0.2%) |
| Occupation, n (%)^4^ |  |  |
| Salaried | 135 (15.2%) | 130 (14.4%) |
| Self-employed | 456 (51.5%) | 478 (53.1%) |
| Housewife | 95 (10.7%) | 84 (9.3%) |
| Student | 9 (1.0%) | 9 (1.0%) |
| Unemployed | 190 (21.4%) | 199 (22.1%) |
| Education level, n (%) ^4^ |  |  |
| None | 169 (19.1%) | 170 (18.9%) |
| Primary | 510 (57.6%) | 518 (57.6%) |
| Secondary | 183 (20.7%) | 188 (20.9%) |
| University/Tertiary | 23 (2.6%) | 24 (2.7%) |
| Study site, n (%) |  |  |
| Lusaka | 539 (60.8%) | 550 (61.1%) |
| Mwanza | 347 (39.2%) | 350 (38.9%) |
| ^1^Lipid Nutritional Supplement; ^2^Lipid Nutritional Supplement with added Vitamins and Minerals; ^3^Standard deviation; ^4^Missing values for: on tuberculosis treatment, 8 (0.9%) LNS arm, 7 (0.8%) LNS-VM arm; marital status: 1 (0.1%) LNS arm; occupation: 1 (0.1%) LNS arm; education level: 1 (0.1%) LNS arm. | | |

| Table S2: Comparison of baseline characteristics between patients included in analysis of serum phosphate at 12 weeks post-ART versus those not included (due to death, lost to follow-up, or lack of data). | | | |
| --- | --- | --- | --- |
|  | Died or lost to follow-up before 12 weeks-post ART (n=1017) | Followed-up at 12 weeks post ART (n=798) | *P*-value^1^ |
| Age years, mean (SD^2^) | 35.1 (9.0) | 36.7 (9.9) | 0.002 |
| Female, n (%) | 482 (47.4%) | 418 (52.4%) | 0.04 |
| On tuberculosis treatment, n (%)^3^ | 149 (14.7%) | 155 (19.4%) | 0.03 |
| Baseline CD4 count cells/ µL, mean (SD) | 128.5 (99.3) | 148.2 (100.4) | <0.001 |
| CD4 count <100 (cells/µL), n (%) | 479 (47.1%) | 311 (39.0%) | 0.001 |
| BMI <17 (kg/m^2^), n (%) | 644 (63.3%) | 430 (53.9%) | <0.001 |
| Social economic status, n (%) |  |  |  |
| Lowest | 227 (22.3%) | 137 (17.2%) | 0.06 |
| Low | 212 (20.9%) | 162 (20.3%) |  |
| Middle | 194 (19.1%) | 168 (21.1%) |  |
| High | 196 (19.3%) | 159 (19.9%) |  |
| Highest | 188 (18.5%) | 172 (21.6%) |  |
| Marital status, n (%)^3^ |  |  |  |
| Married | 475 (46.7%) | 378 (47.4%) | 0.05 |
| Widow/widower | 102 (10.0%) | 101 (12.7%) |  |
| Divorced/separated | 305 (30.0%) | 205 (25.7%) |  |
| Single | 129 (12.7%) | 114 (14.3%) |  |
| Lives with partner | 5 (0.5%) | 0 (0.0%) |  |
| Occupation, n (%)^3^ |  |  |  |
| Salaried | 155 (15.2%) | 116 (14.5%) | 0.20 |
| Self-employed | 545 (53.6%) | 400 (50.1%) |  |
| Housewife | 97 (9.5%) | 86 (10.8%) |  |
| Student | 6 (0.6%) | 12 (1.5%) |  |
| Unemployed | 213 (20.9%) | 184 (23.1%) |  |
| Education level, n (%) ^3^ |  |  |  |
| None | 207 (20.4%) | 136 (17.0%) | 0.37 |
| Primary | 579 (57.0%) | 466 (58.4%) |  |
| Secondary | 204 (20.1%) | 173 (21.7%) |  |
| University/Tertiary | 26 (2.6%) | 23 (2.9%) |  |
| Study site, n (%) |  |  |  |
| Lusaka | 591 (58.1%) | 520 (65.2%) | 0.002 |
| Mwanza | 426 (41.9%) | 278 (34.8%) |  |
| ^1^Comparison done by t-test for continuous variables and chi-squared test for categorical variables. ^2^Standard deviation; ^3^Missing values for: on tuberculosis treatment, 9 (0.9%) died or lost to follow-up group, 6 (0.8%) completed follow-up group; marital status: 1 (0.1%) died or lost to follow-up group; occupation: 1 (0.1%) died or lost to follow-up group; education level: 1 (0.1%) died or lost to follow-up group. | | | |

| Table S3: Comparison of baseline characteristics between patients included in analysis of serum potassium at 12 weeks post-ART versus those not included (due to death, lost to follow-up, or lack of data). | | | |
| --- | --- | --- | --- |
|  | Died or lost to follow-up before 12 weeks post ART (n=1061) | Followed-up at 12 weeks post ART (n=754) | *P*-value^1^ |
| Age years, mean (SD^2^) | 35.0 (9.0) | 37.0 (9.9) | <0.001 |
| Female, n (%) | 504 (47.5%) | 396 (52.5%) | 0.04 |
| On tuberculosis treatment, n (%)^3^ | 152 (14.3%) | 152 (20.2%) | 0.004 |
| Baseline CD4 count cells/ µL, mean (SD) | 129.1 (99.1) | 148.5 (100.8) | <0.001 |
| CD4 count <100 (cells/µL), n (%) | 497 (46.8%) | 293 (38.9%) | 0.001 |
| BMI <17 (kg/m^2^), n (%) | 669 (63.1%) | 405 (53.7%) | <0.001 |
| Social economic status, n (%) |  |  |  |
| Lowest | 236 (22.2%) | 128 (17.0%) | 0.03 |
| Low | 218 (20.6%) | 156 (20.7%) |  |
| Middle | 201 (18.9%) | 161 (21.4%) |  |
| High | 212 (20.0%) | 143 (19.0%) |  |
| Highest | 194 (18.3%) | 166 (22.0%) |  |
| Marital status, n (%)^3^ |  |  |  |
| Married | 497 (46.8%) | 356 (47.2%) | 0.06 |
| Widow/widower | 104 (9.8%) | 99 (13.1%) |  |
| Divorced/separated | 314 (29.6%) | 196 (26.0%) |  |
| Single | 140 (13.2%) | 103 (13.7%) |  |
| Lives with partner | 5 (0.5%) | 0 (0.0%) |  |
| Occupation, n (%)^3^ |  |  |  |
| Salaried | 166 (15.7%) | 105 (13.9%) | 0.43 |
| Self-employed | 547 (51.6%) | 398 (52.8%) |  |
| Housewife | 104 (9.8%) | 79 (10.5%) |  |
| Student | 7 (0.7%) | 11 (1.5%) |  |
| Unemployed | 236 (22.2%) | 161 (21.4%) |  |
| Education level, n (%) ^3^ |  |  |  |
| None | 214 (20.2%) | 129 (17.1%) | 0.44 |
| Primary | 598 (56.4%) | 447 (59.3%) |  |
| Secondary | 221 (20.8%) | 156 (20.7%) |  |
| University/Tertiary | 27 (2.5%) | 22 (2.9%) |  |
| Study site, n (%) |  |  |  |
| Lusaka | 648 (61.1%) | 463 (61.4%) | 0.89 |
| Mwanza | 413 (38.9%) | 291 (38.6%) |  |
| ^1^comparison done by t test for continuous variables and chi-squared test for categorical variables. ^2^ Standard deviation; ^3^Missing values for: on tuberculosis treatment, 8 (0.8%) died or lost to follow-up group, 7 (0.9%) completed follow-up group; marital status: 1 (0.1%) died or lost to follow-up group; occupation: 1 (0.1%) died or lost to follow-up group; education level: 1 (0.1%) died or lost to follow-up group. | | | |

Table S4a: Poisson regression hazard ratios (HR) for time varying serum electrolyte values associated with mortality in the LNS-VM arm

| Current value | Change from previous value | N deaths | 100 py | Mortality rate per 100 py | Unadjusted HR^1^ (95% CI) | p-value | Adjusted^2^ HR (95% CI) | p-value |
| --- | --- | --- | --- | --- | --- | --- | --- | --- |
| *Phosphate^5^* |  |  |  |  |  |  |  |  |
| Low | Decrease | 18 | 0.18 | 102 (64, 162) | 6.32 (3.18, 12.58) |  | 3.54 (1.70, 7.35) |  |
| Low | No change | 9 | 0.24 | 37 (19, 71) | 1.91 (0.84, 4.37) |  | 1.89 (0.79, 4.55) |  |
| Low | Increase | 1 | 0.01 | 148 (21, 1050) | 13.57 (1.78, 103.71) |  | 22.93 (2.57, 204.31) |  |
| Middle | Decrease | 15 | 0.18 | 82 (49, 136) | 5.94 (2.88, 12.23) |  | 3.39 (1.56, 7.37) |  |
| Middle | No change | 15 | 0.83 | 18 (11, 30) | Reference | <0.001 | Reference | 0.002 |
| Middle | Increase | 14 | 0.13 | 112 (66, 189) | 7.33 (3.49, 15.38) |  | 5.61 (2.48, 12.67) |  |
| High | Decrease | 2 | 0.02 | 84 (21, 337) | 4.39 (0.99, 19.44) |  | 2.77 (0.59, 13.06) |  |
| High | No change | 9 | 0.22 | 40 (21, 78) | 2.27 (0.99, 5.18) |  | 1.78 (0.71, 4.49) |  |
| High | Increase | 19 | 0.23 | 84 (54, 132) | 4.68 (2.37, 9.26) |  | 2.27 (1.10, 4.66) |  |
|  |  |  |  |  |  |  |  |  |
| *Potassium^6^* |  |  |  |  |  |  |  |  |
| Low | - | 46 | 0.43 | 108 (81, 144) | 2.25 (1.45, 3.49) |  | 1.36 (0.85, 2.19) |  |
| Middle | - | 49 | 1.43 | 34 (26, 45) | Reference | <0.001 | Reference | 0.22 |
| High | - | 32 | 0.43 | 75 (53, 104) | 1.74 (1.07, 2.82) |  | 1.49 (0.89, 2.51) |  |
| - | Decrease | 48 | 0.48 | 100 (76, 133) | 2.65 (1.69, 4.18) |  | 1.51 (0.92, 2.50) |  |
| - | No change | 39 | 1.31 | 30 (22, 41) | Reference | <0.001 | Reference | 0.27 |
| - | Increase | 39 | 0.47 | 82 (60, 112) | 2.39 (1.47, 3.88) |  | 1.19 (0.70, 2.00) |  |

^1^ For phosphate low is 0.04-0.96, middle is 0.97-1.44, high is 1.45-5.27. For potassium low is 0.1-3.63, middle is 3.63-4.5 and high is 4.6-10 mmol/L.

^2^ For serum phosphate daily changes were defined as: <-0.02 a decrease, (-0.02, 0.02) no change and >0.02 increase. For potassium daily changes were defined as <-0.03 a decrease, (-0.03, 0.03) no change and >0.03 an increase.

^3^ A Lexis expansion for time was adjusted for in four-week time bands, both pre-ART and post ART. Being on ART was also adjusted for.

^4^ Adjusted for trial arm (LNS or LNS-VM), time band (as for ^3^), country, sex, age group (18-29, 30-39, 40-49, ≥50), baseline CD4 count (<50, 50-99, 100-199, ≥200 cells/μl), body mass index (continuous), baseline TB treatment (yes/no), baseline oedema (y/n), baseline CRP (<10, 10-49, 50-159, ≥160 mg/l).

^5^ P-value for interaction between current level and change from previous level unadjusted model = 0.34, adjusted model = 0.08

^6^ P-value for interaction between current level and change from previous level unadjusted model = 0.45, adjusted model = 0.16.

Table S4b: Poisson regression hazard ratios (HR) for time varying serum electrolyte values associated with mortality in the LNS arm

| Current value | Change from previous value | N deaths | 100 py | Mortality rate per 100 py | Unadjusted HR^1^ (95% CI) | p-value | Adjusted^2^ HR (95% CI) | p-value |
| --- | --- | --- | --- | --- | --- | --- | --- | --- |
| *Phosphate* |  |  |  |  |  |  |  |  |
| Low | Decrease | 6 | 0.17 | 36 (16, 81) | 1.61 (0.65, 3.95) |  | 0.97 (0.36, 2.61) |  |
| Low | No change | 11 | 0.24 | 46 (26, 84) | 1.79 (0.87, 3.67) |  | 1.92 (0.88, 4.17) |  |
| Low | Increase | 3 | 0.02 | 192 (62, 596) | 10.67 (3.17, 35.87) |  | 5.46 (1.42, 21.01) |  |
| Middle | Decrease | 16 | 0.16 | 97 (60, 159) | 4.32 (2.28, 8.20) |  | 2.34 (1.17, 4.69) |  |
| Middle | No change | 23 | 0.84 | 27 (18, 41) | Reference | <0.001 | Reference | 0.03 |
| Middle | Increase | 13 | 0.15 | 86 (50, 148) | 3.27 (1.64, 6.54) |  | 1.62 (0.75, 3.50) |  |
| High | Decrease | 3 | 0.02 | 132 (42, 408) | 4.83 (1.43, 16.25) |  | 5.29 (1.41, 19.84) |  |
| High | No change | 5 | 0.20 | 26 (11, 61) | 0.89 (0.34, 2.33) |  | 1.02 (0.37, 2.78) |  |
| High | Increase | 9 | 0.16 | 55 (29, 105) | 2.23 (1.03, 4.84) |  | 2.30 (1.01, 5.24) |  |
|  |  |  |  |  |  |  |  |  |
| *Potassium* |  |  |  |  |  |  |  |  |
| Low |  | 47 | 0.48 | 97 (73, 129) | 1.95 (1.29, 2.94) |  | 1.05 (0.67, 1.64) |  |
| Middle |  | 55 | 1.37 | 40 (31, 52) | Reference | <0.001 | Reference | 0.92 |
| High |  | 21 | 0.36 | 59 (38, 90) | 1.28 (0.74, 2.20) |  | 1.12 (0.64, 1.97) |  |
|  | Decrease | 49 | 0.46 | 106 (80, 140) | 2.78 (1.77, 4.34) |  | 1.31 (0.81, 2.13) |  |
|  | No change | 39 | 1.29 | 30 (22, 41) | Reference | <0.001 | Reference | 0.33 |
|  | Increase | 34 | 0.43 | 79 (56, 110) | 2.36 (1.44, 3.87) |  | 1.44 (0.87, 2.38) |  |

^1^ For phosphate low is 0.04-0.96, middle is 0.97-1.44, high is 1.45-5.27. For potassium low is 0.1-3.63, middle is 3.63-4.5 and high is 4.6-10 mmol/L.

^2^ For serum phosphate daily changes were defined as: <-0.02 a decrease, (-0.02, 0.02) no change and >0.02 increase. For potassium daily changes were defined as <-0.03 a decrease, (-0.03, 0.03) no change and >0.03 an increase.

^3^ A Lexis expansion for time was adjusted for in four-week time bands, both pre-ART and post ART. Being on ART was also adjusted for.

^4^ Adjusted for trial arm (LNS or LNS-VM), time band (as for ^3^), country, sex, age group (18-29, 30-39, 40-49, ≥50), baseline CD4 count (<50, 50-99, 100-199, ≥200 cells/μl), body mass index (continuous), baseline TB treatment (yes/no), baseline oedema (y/n), baseline CRP (<10, 10-49, 50-159, ≥160 mg/l).

^5^ P-value for interaction between current level and change from previous level unadjusted model = 0.03, adjusted model = 0.04.

^6^ P-value for interaction between current level and change from previous level unadjusted model = 0.41, adjusted model = 0.70.

Figure S1: Changes in predicted mean serum phosphate over time by trial arm, for an individual who started ART three weeks after recruitment and who survived to the end of the study

Figure S2: Changes in predicted mean serum potassium over time by trial arm, for an individual who started ART three weeks after recruitment and who survived to the end of the study

Figure S3: Changes in predicted mean serum phosphate over time by trial arm

1. for an individual who started ART one week after recruitment
2. for an individual who started ART two weeks after recruitment
3. for an individual who started ART four weeks after recruitment

Figure 3a)

Figure 3b)

Figure 3c)

Figure S4: Changes in predicted mean serum potassium over time by trial arm

1. for individuals who started ART one week after recruitment
2. for individuals who started ART two weeks after recruitment
3. for individuals who started ART four weeks after recruitment

Figure 4a)

Figure 4b)

Figure 4c)
